# Towards rare-earth-free white light-emitting diode devices based on the combination of dicyanomethylene and pyranine as organic dyes supported on zinc single-layered hydroxide

**DOI:** 10.3762/bjnano.10.75

**Published:** 2019-03-25

**Authors:** Jeff L Nyalosaso, Rachod Boonsin, Pierre Vialat, Damien Boyer, Geneviève Chadeyron, Rachid Mahiou, Fabrice Leroux

**Affiliations:** 1 Université Clermont Auvergne, CNRS, SIGMA Clermont, ICCF, F-63000 Clermont–Ferrand, France

**Keywords:** dicyanomethylene (DCM), hybrid luminescent films, light-emitting diode (LED), pyranine, zinc hydroxyacetate

## Abstract

A new luminescent composite film resulting from the dispersion of luminescent organic dyes in a single-layered hydroxide (SLH)-type inorganic matrix has been developed. Two fluorescent organic dyes emitting visible light upon blue LED excitation were investigated in this study: dicyanomethylene (DCM) and pyranine (HPTS). These dyes exhibit broad emission bands that cover a large part of the visible spectrum. The concept developed in our work consisted in keeping SLH in its wet form to ensure a good dispersion of the fluorescent dyes prior to immobilizing the hybrid materials in a silicone polymer to achieve luminescent composite films. We demonstrate that these coatings stacked upon each other and placed above a blue LED lead to white-light emission with suitable photometric parameters for applications in lighting or display devices: colour temperature of 5409 K and colour rendering index (CRI) of 81.

## Introduction

Light-emitting diode (LED) devices are the most developed lighting systems today. 95% of LEDs found on the market generate white light by combining the blue light of a semiconductor diode (GaN or InGaN) with the broad yellow emission of the Y_3_Al_5_O_12_:Ce^3+^ (YAG:Ce) phosphor. The use of a phosphor is essential since, to date, no semiconductor diode has been found to emit directly into the white. The system YAG:Ce/blue LED gives a low colour-rendering index (CRI < 80) and a high correlated colour temperature (CCT > 5000 K), which requires the addition of expensive and moisture-sensitive red phosphors (fluoride or nitride) [1]. These phosphors involve rare-earth-doped inorganic phosphors that are extensively used in high-technology devices, LED lighting, mobile phones, flat panel display and wind turbines.

While a series of breakthroughs and advances have been made for efficient blue LEDs, currently the research on white LEDs is mainly focused on the development of rare-earth-free phosphors [2–3]. The worldwide demand for rare-earth elements (REEs), especially within the sectors of renewable energy, military, and consumer electronics, is projected to reach 200.000 tons per year by 2025 while the current annual supply is estimated to be only 113.000 tons. The attempt to increase the REE supply at a rate sufficiently high to meet the increase in demand faces economic, political and environmental limitations [4–5]. Over 95% of the global REE supply comes from China, which also has the largest demand for REEs at 65% of the total demand, even before the United States (15%). In 2010, China announced a 40% reduction in exports of REEs as it wants to reduce stress on its REE reserves. This led to a 600% price increase [6]. Moreover, the cost of a white LED lamp is strongly linked to the price of REEs as the latter represents 12% of the total.

The explosion of demand combined with a monopolistic supply source represents a real risk for the deployment of LED technology in the years to come. Developing rare-earth-free phosphors is therefore a major issue. In this context, it seems crucial to identify new cheap and REE-free phosphors capable of delivering cost-effective light energy conversion.

Luminescent organic dyes are a relevant alternative to REEs. Indeed, they are known to exhibit high luminous efficiency at low cost and may be associated with commercial blue or UV LEDs to generate coloured light and white light. However, most of them exhibit luminescence properties only in liquid solution when any aggregation-induced quenching is prevented. But we have shown recently that the dyes can exhibit fluorescence in the solid state when they are dispersed in an inorganic matrix such as silica [7]. Studies have been carried out on materials obtained by mixing a dye and a double-layered hydroxide solid compound [8–11]. Unfortunately, the optical properties of these materials, particularly their performance, are not yet satisfactory.

For all these reasons, we have studied the development of a rare-earth-free LED device by considering the use of stabilized synthetic organic dyes in an inorganic solid matrix to form a luminescent hybrid material. Some of these hybrid materials consist of a layered inorganic matrix, the role of which is to trap the organic molecules by intercalation so as to preserve their optical properties [10–11]. In the solid matrix the molecule dyes are arranged in the interlayer spaces by monolayer particle assembly and a direct anion-exchange procedure in organic media [12]. Depending on the nature of the layers, one can have structures of one, two or three dimensions [13].

Single-layered hydroxides (SLH), prepared by the polyol method [14–15], are part of the one-dimensional structures. Their general formula is M(OH)_2−_*_y_*X*_y_*·*n*H_2_O in which M represents a cation of a divalent transition metal such as Zn^2+^, Ni^2+^, Co^2+^ and Cu^2+^ and X represents an interfoliary anion such as acetate or nitrate.

Similar to zinc basic salts, zinc hydroxyacetate Zn_5_(OH)_8_(CH_3_COO)_2_·2H_2_O (Zn-SLH) is a white solid that is suitable for our study. Thanks to its positive lamellar charge, Zn-SLH can serve as a diluting solid allowing for the stabilization of negatively charged molecules or molecules with electron donor sites, i.e., double bonds [11].

The luminescent organic dye is selected so as to ensure good compatibility with the SLH compound and satisfy the synthesis conditions of the latter. It must therefore (i) exhibit negative charges or electron donor sites in order to interact with the positively charged SLH compound; (ii) be water-soluble in the reaction medium (generally water, ethanol or polyol); and (iii) be optically active at basic pH values, for which the formation of the SLH compound is favoured.

**Figure 1:**
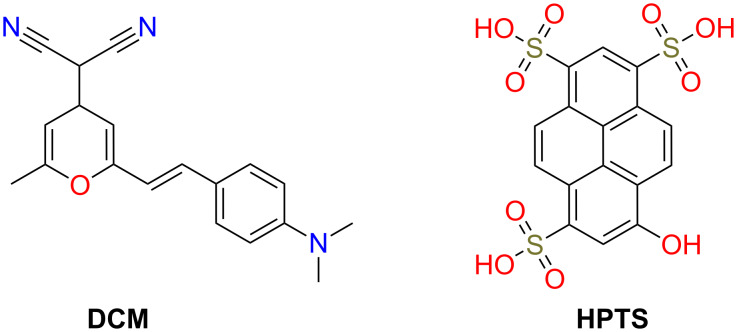
Dye molecules used for the preparation of the organic phosphor film.

Among the organic dyes that meet these criteria, there are xanthene derivatives (fluorescein, rhodamine), acriflavines, arylsulfonates, cyanines and pyrans. Only a few organic dyes can be excited by a blue LED emission centred at 450 nm. Among these dyes, mention can be made of dicyanomethylene and pyranine (Figure 1).

**Figure 2:**
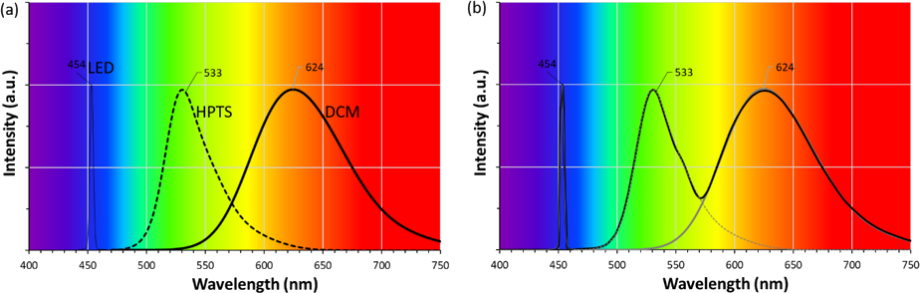
(a) Normalized emission spectra of blue LED light and selected dyes in ethanol solution: DCM and HPTS and (b) their predicted combined spectrum.

Dicyanomethylene (4-(dicyanomethylene)-2-methyl-6-(4-dimethylaminostyryl)-4*H*-pyran, DCM) belongs to the pyran family (heterocycles in which five carbon atoms and one oxygen atom are present in the ring structure) while pyranine (trisodium 8-hydroxypyrene-1,3,6-trisulfonate, HPTS) belongs to the family of arylsulfonates. The first one exhibits, in ethanol solution, red emission with a maximum at 624 nm after excitation at 450 nm and the second one is water-soluble and characterized by a yellow-green emission with a maximum at 533 nm after excitation at 450 nm. These organic dyes can be combined with the emission of a commercial blue LED (Figure 2a).

The emission spectrum of HPTS ranges from 465 to 625 nm, while that of DCM is wider and extends from 520 to 750 nm. By coupling their spectra with that of a blue LED (λ_max_ = 450 nm), we can expect a wider coverage of the visible spectrum that can generate white light. The combination of these two dyes is therefore of interest for the realization of white LEDs (WLEDs) (Figure 2b).

The chemical bond between these organic dyes and the Zn-SLH matrix should provide a good dispersion of the luminescent material in the silicone matrix while preventing at the same time the organic dyes from aggregation [16]. Indeed, the aggregation of organic molecules leading to the quenching of their fluorescence occurs when the hybrid compound (SLH-Dye) is prepared in the dry solid state. The originality of our preparation process lies in the dispersion of the organic dyes in the SLH matrix in its wet form. The resulting product consisting of dispersed hybrid sheets is then immobilized and dried in a polymer network such as silicone, so as to form a luminescent composite film. Another method reported in the literature to overcome the aggregation-induced quenching mechanism consists in modifying the structure of the organic dyes with units such as tetraphenylethene (TPE) in order to obtain aggregation-induced emission (AIE) mechanism [17].

The structural and morphological properties of Zn-SLH have been studied in order to highlight its lamellar structure. The optical properties of the luminescent wet hybrid materials and films were recorded. Finally, a down-conversion pseudo-white LED was designed by simply associating, in remote-phosphor configuration, silicone/SLH-dye composite film and a GaN LED emitting at 450 nm. Composite films with a single dye, HPTS or DCM, were tested as well as the combination of these two materials. Finally, the photometric parameters of these systems have been investigated under LED excitation.

## Results and Discussion

### Structural properties of Zn-SLH

**Figure 3:**
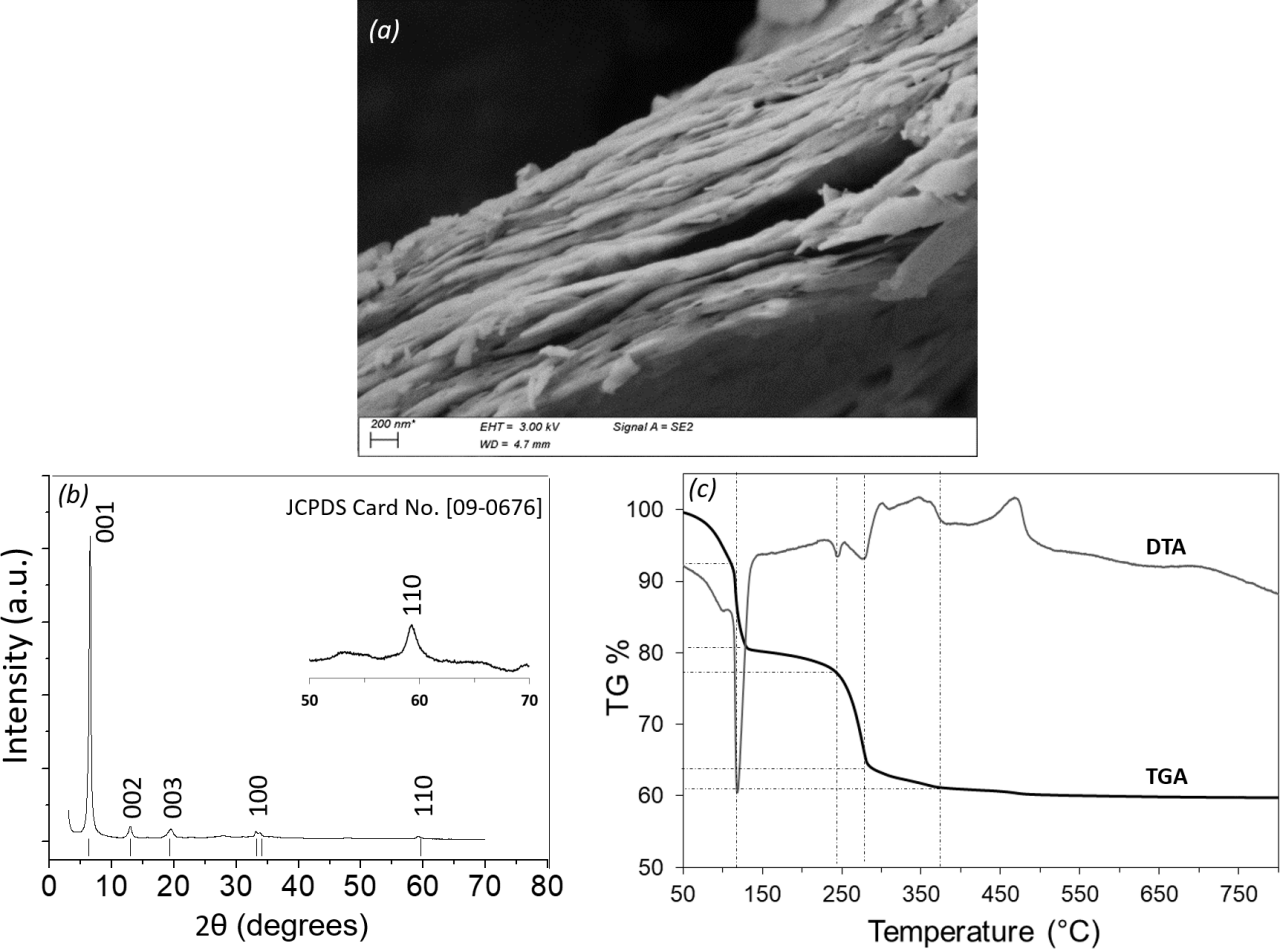
(a) SEM image, (b) XRD pattern and (c) TGA/DTA of zinc hydroxyacetate single-layered hydroxide.

The prepared Zn-SLH in the dry state is a white powder which, in a suspension medium, has a filamentous appearance reminding lamellar structures. Scanning electronic microscopy (Figure 3a) reveals that Zn-SLH compounds are aggregated sheets without any defined and regular shape.

Figure 3b shows the powder XRD pattern of the dried Zn-SLH, similar to the one reported by Poul and co-workers [14]. The most intense (001) peak at 2θ = 6.61° corresponds to an interlayer *d*-spacing of 1.33 nm where the intercalation of acetate anions occurs [1]. Such intense (001) reflections are characteristic of a layered structure. The other reflections, *hkl* with *h* or *k* ≠ 0, are much weaker and exhibit usual asymmetrical enlargement for disordered pillared compounds [18]. Second-order (002) and third-order (003) peaks correspond to *d*-spacings of 0.68 nm and 0.44 nm, respectively. Moreover, the presence of the (110) diffraction line at 2θ = 59.3° is attributed to OH-edge-sharing platelets based on Zinc cations [18]. The XRD data evidenced the presence of a single phase with the following lattice parameters: *a* = 0.312 nm, *c* = 3.98 nm and *d* = 1.33 nm.

Structures of SLHs have been described by authors such as Rogez and co-workers [18]. The structure derived from botallackite or brucite consists of a quasi-planar triangular array of octahedral divalent metal ions separated by anions, e.g., acetate. Those anions coordinate the metal atoms and water molecules. The intercalation of new guest molecules or ions that substitute the acetate anions located in the interlayer space brings small variations in the molecular area of each metal atom.

TG-DTA measurements (Figure 3c) carried out on the dried Zn-SLH showed mass losses of 39% upon heating to 800 °C through the decomposition of zinc hydroxyacetate up to the fully inorganic ZnO. The associated thermal events are well documented [19] and correspond to the loss of two water molecules below 100 °C and four additional molecules below 250 °C, together with acetone and CO_2_ as well as acetic anhydride to finally form ZnO. The TG-DTA data were used to verify the chemical formula of the compound Zn_5_(OH)_8_(CH_3_COO)_2_·*n*H_2_O. The mass formula at room temperature is related to *M*(RT) = 5·ZnO/(1 − 0.39) leading to 667.29 g·mol^−1^ (per 5·Zn) and corresponding to a hydration rate of *n* = 4.79, much higher than the value of 2 that is usually reported. It may be explained by a sample largely hydrated with weakly bonded water molecules as can be inferred from the mass loss between 50 and 100 °C. The theoretical mass loss of 4.79·H_2_O represents 12.92% while the loss of two acetate anions is 17.68%. These values are smaller than the measured mass loss. The mass loss below 150 °C is not only due to the evaporation of water molecules, some dehydroxylation may also occur.

### Photoluminescence properties of the integrated dyes

Absolute quantum yield (QY_ab_) is defined as the efficiency at which a given material re-emits by fluorescence a certain number of photons absorbed at a given wavelength. The parameters measured in a 3.3 inch integrating sphere provide information on the internal conversion (QY_int_) and the absorbance (Abs) of the studied material. QY_int_ is defined as the ratio between photons emitted and photons absorbed by the material upon external excitation. The product of these two parameters gives the absolute quantum yield in percentage (QY_ab_ = QY_int_ × Abs × 100%). Measuring the absolute quantum yield (through the scan mode of the software system) as a function of the excitation wavelength is used to plot the profile [QY_ab_ = *f*(λ_ex_)] from which one can determine at which excitation wavelength the maximum of QY_ab_ occurs. QY_ab_ has a direct impact on the photoluminescence performance of the studied material. A fraction of the excitation light will be absorbed by the dye molecule, internally converted, transferred and emitted at a longer wavelength.

**Figure 4:**
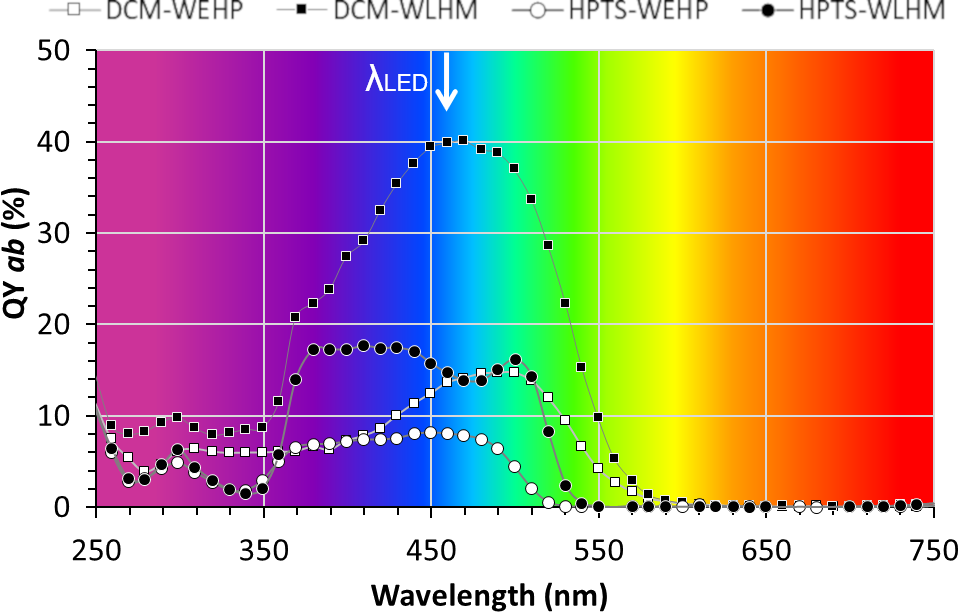
Photoluminescence excitation spectra. Absolute quantum yield (QY_ab_) as a function of the excitation wavelength for DCM and HPTS in the wet hybrid compounds (WLHM) and in the silicon-based film (WEHP).

Figure 4 illustrates the recording of the evolution of photoluminescence QY_ab_ as a function of excitation wavelength for each wet luminescent hybrid material (WLHM) and white-emitting hybrid phosphor film (WEHP).

**Figure 5:**
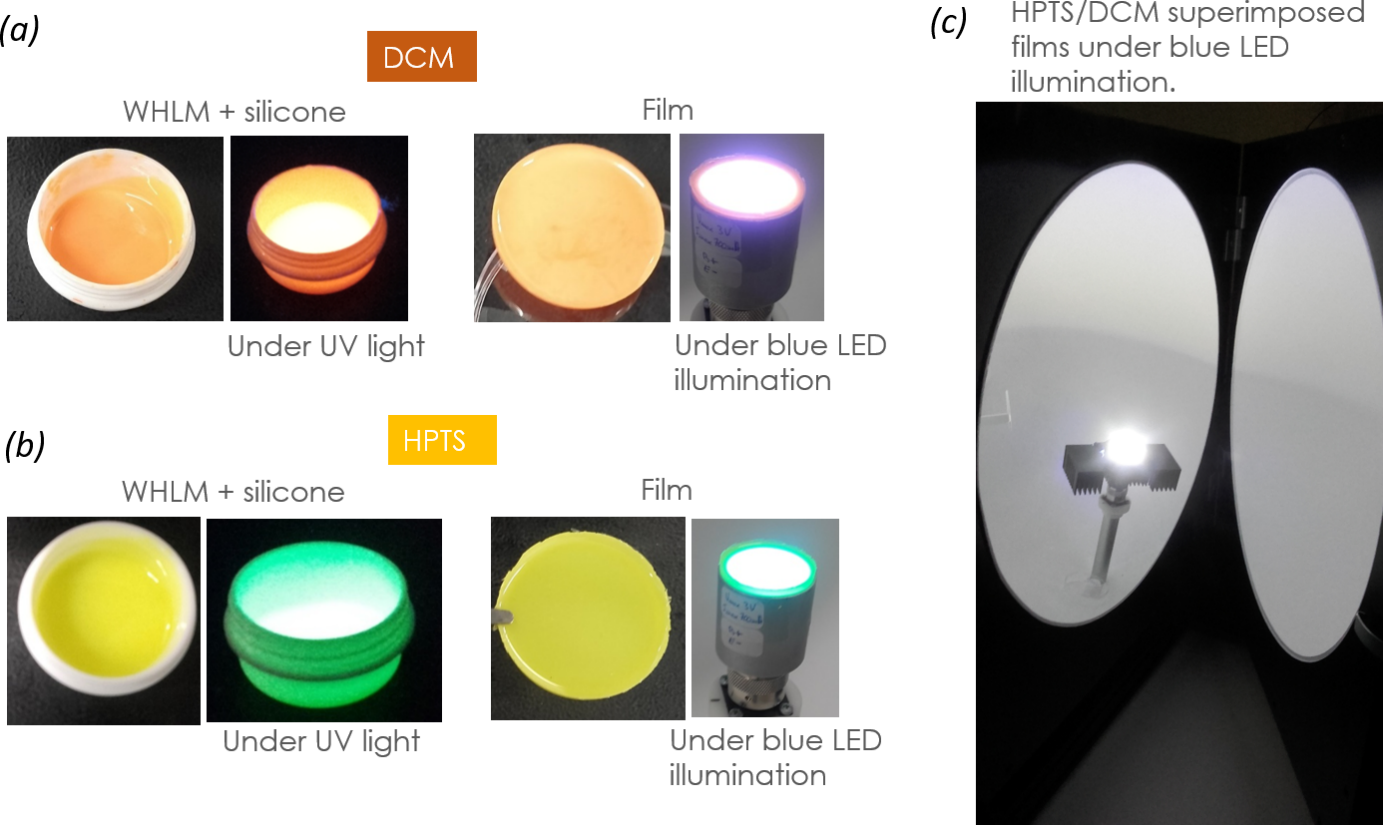
Photographs of (a) DCM- and (b) HPTS-based hybrid materials in the wet mixture containing pre-formed silicon film (with and without UV irradiation) and in the form of the completely formed film (with and without blue-LED illumination). (c) Photo of the two films superimposed (HPTS on DCM) under blue-LED illumination in the opened integrating sphere.

Thus, we have the value of QY_ab_ for a given excitation wavelength, in our case the one of the blue LED (450 nm). It can be seen that the organic dyes begin to be excited significantly at an excitation wavelength of 350 nm (near UV, see photographs of the fluorescent WEHP materials under UV light in Figure 5a,b). Then, they reach their maximum QY_ab_ in the region between 370 and 510 nm, mainly in the visible region.

The QY_ab_ of these dyes in both WLHM and WEHP films were measured at the wavelength of interest and the values are given in Table 1. The maximum values of QY_ab_ were measured at λ_exc_ 470 nm and 500 nm for DCM (40.2%) and HPTS (16.2%) dyes in their WLHM form, respectively. By moving from the wet state to the film form, QY_ab_ is reduced by more than 63% and 45% for DCM and HPTS dyes, respectively.

**Table 1:** Absolute quantum yield (QY_ab_) values of DCM and HPTS dyes in the WLHMs and in WEHP films, excited at 450 nm. The excitation wavelength values corresponding to the highest values of QY_ab_ of each compound are given.

	DCM	HPTS

QY_ab_ in WLHM (%)	40.0	15.0
QY_ab_ max at λ (nm)	40.2% @ 470 nm	16.2% @ 500 nm
QY_ab_ in silicon film (%)	13.6	8.1
QY_ab_ max at λ (nm)	14.7% @ 500 nm	8.1 @ 450 nm

When excited, the organic dyes studied here exhibit strong fluorescence in diluted solution. In our study, these dyes were embedded in the inorganic Zn-SLH matrix in the wet state, before being immobilized in a dried silicone-based film.

It should be noted that the WLHM samples can distort the interpretation of QY_ab_ insofar as a part of dye that is in solution (free dyes existing in the wet compound) would dictate its photoluminescence properties (leading to higher QY_ab_ values), therefore masking those intrinsic to the solid (dyes actually supported on Zn-SLH). The measurements of QY_ab_ on the films are more representative of the intrinsic properties of supported dyes.

We focused our attention on the value of QY_ab_ at the excitation wavelength of 450 nm in order to assess the fluorescence of the organic dyes when they are placed on the LED that emits at this value. The films displayed QY_ab_ values of 13.6% and 8.1% for DCM and HPTS dyes, respectively. These QY_ab_ values are relatively weak but should be enough to convert a portion of the blue light into white light.

**Figure 6:**
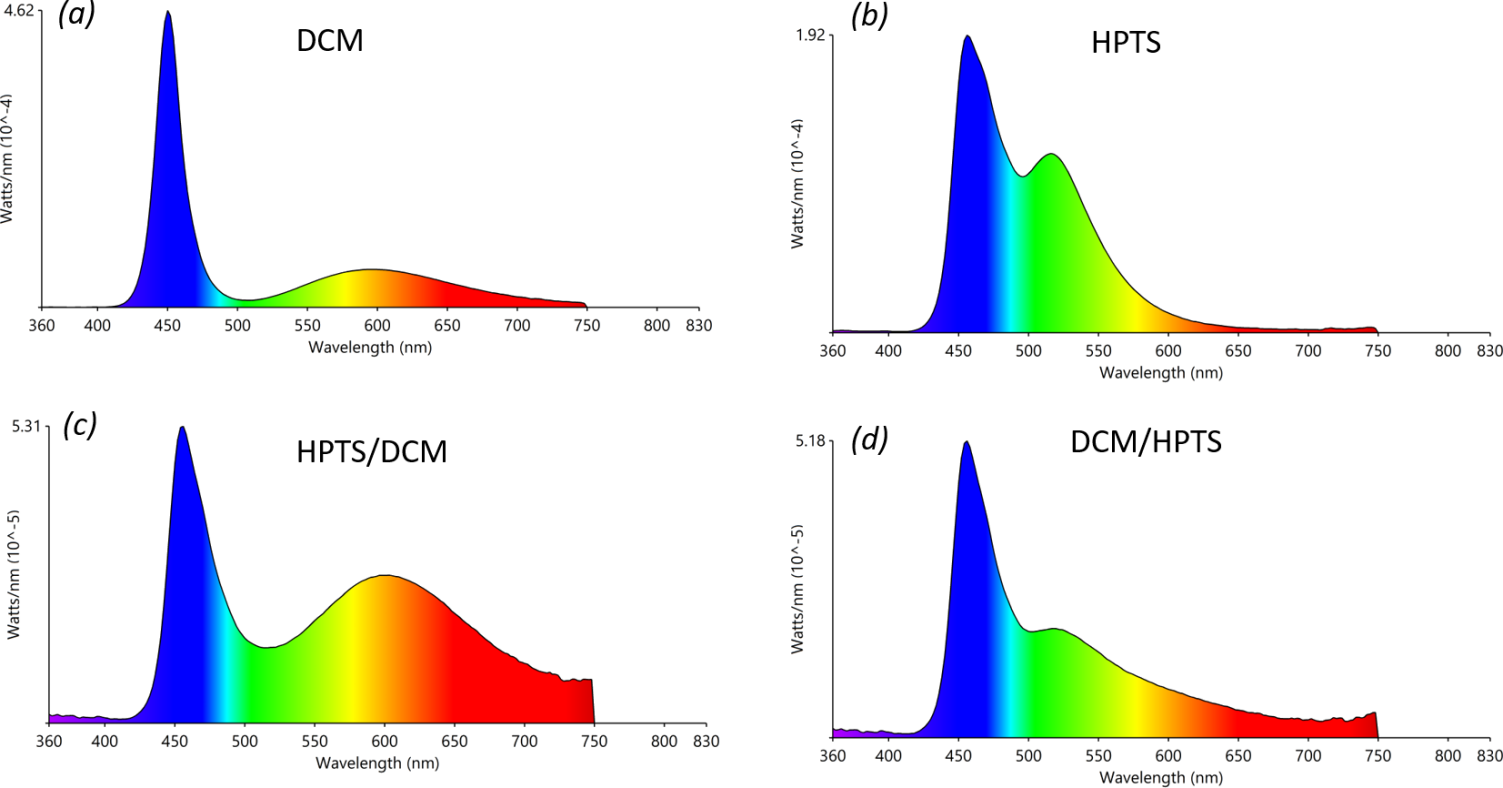
Emission spectra of the silicon films containing: (a) DCM, (b) HPTS, (c) HPTS over DCM and (d) DCM over HPTS; measured under 450 nm LED illumination (440 mA, 3 V). All films were placed in a remote-phosphor configuration at 0.3 cm from the LED chip.

The emission spectra of each WEHP film placed on a 450 nm commercial LED in remote-phosphor configuration are shown in Figure 6. Taken individually, DCM and HPTS dyes have each different profile. We first observed the dominant emission band of the blue LED. Then, in the case of the DCM dye, the emission band, of low intensity, covers a wide visible range from 500 to 750 nm with a maximum at 600 nm (orange). In the case of HPTS dye, the emission band is centred at 517 nm (green).

When the films are stacked upon each other, the corresponding emission spectra represent the emission/excitation/re-emission phenomena between the films. Depending on the stacking order of the films, HPTS above DCM (HPTS/DCM) or DCM above HPTS (DCM/HPTS), the final emission spectra are different.

In the HPTS/DCM/LED configuration, the resulting emission spectrum resembles that of sole DCM but with an increase in the intensity in the wavelength range between 500 and 750 nm. In this configuration, the blue light will first excite the DCM dye-based film. The resulting fluorescence, which covers a wide visible range, will then excite the HPTS dye-based film. In this configuration, all visible colours are emitted relatively evenly.

In the other configuration, DCM/HPTS/LED, the fluorescence resulting from the interaction between the blue light of the LED and the HPTS dye-based film (the emission spectrum of which unevenly covers the visible region) will excite the DCM dye-based film. The resulting emission spectrum strongly resembles that of sole HPTS with a decrease in the contribution of green light.

We can otherwise interpret these results by the difference of QY_ab_ between that of the DCM film (14.7%) and that of HPTS (8.1%). It is therefore convenient to excite first the film that exhibits the strongest QY_ab_.

**Figure 7:**
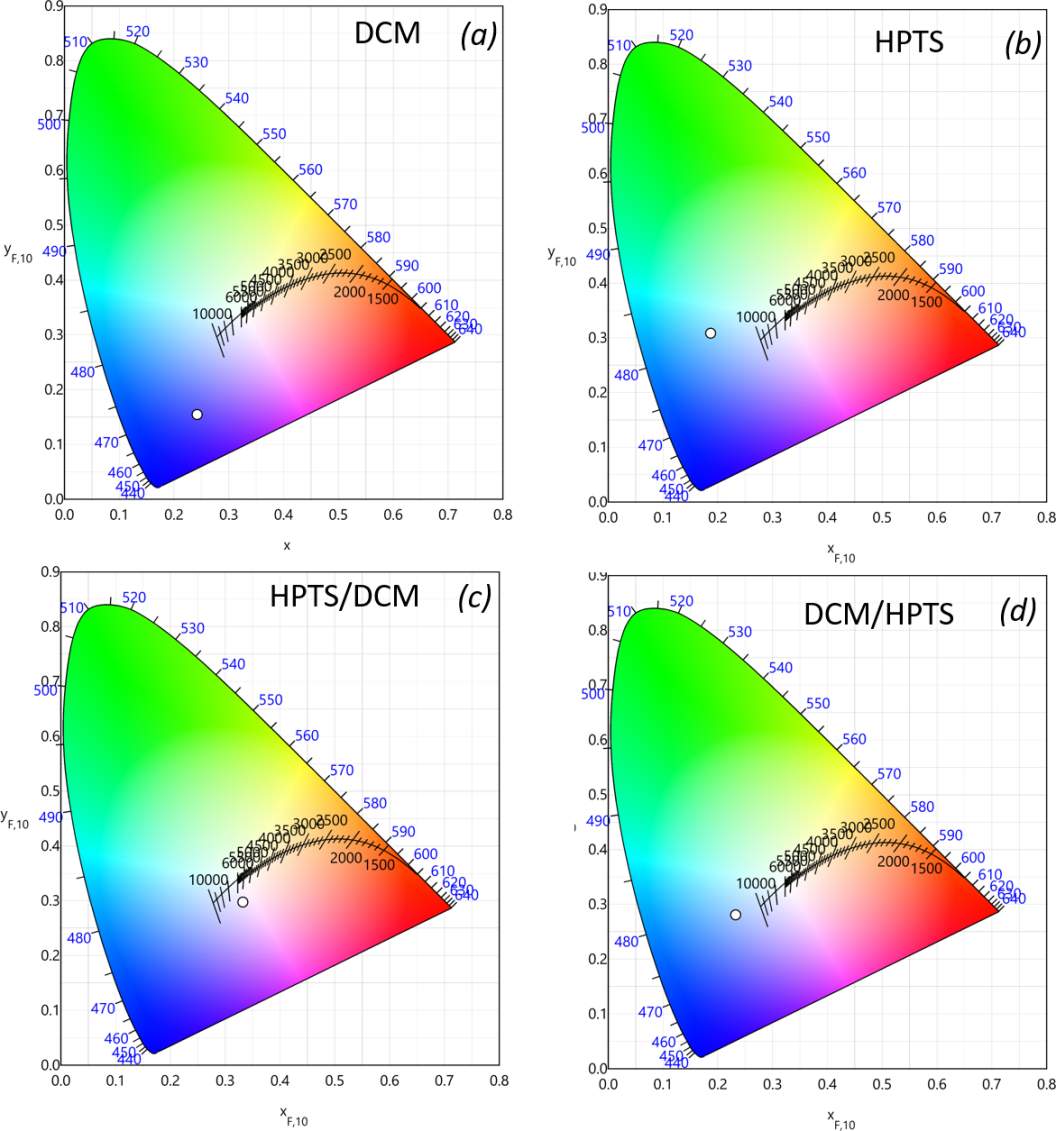
CIE chromaticity diagrams of films containing (a) DCM, (b) HPTS, (c) HPTS over DCM and (d) DCM over HPTS; measured under 450 nm LED illumination (440 mA, 3 V). All films were placed in a remote-phosphor configuration at 0.3 cm from the LED chip.

The normalized two-dimensional colour coordinate systems (chromaticity) of the measured films are shown in Figure 7. The colour points of each film and their superimposition were defined according to the convention of the CIE (Commission Internationale de l’Éclairage, International Commission on Illumination).

**Figure 8:**
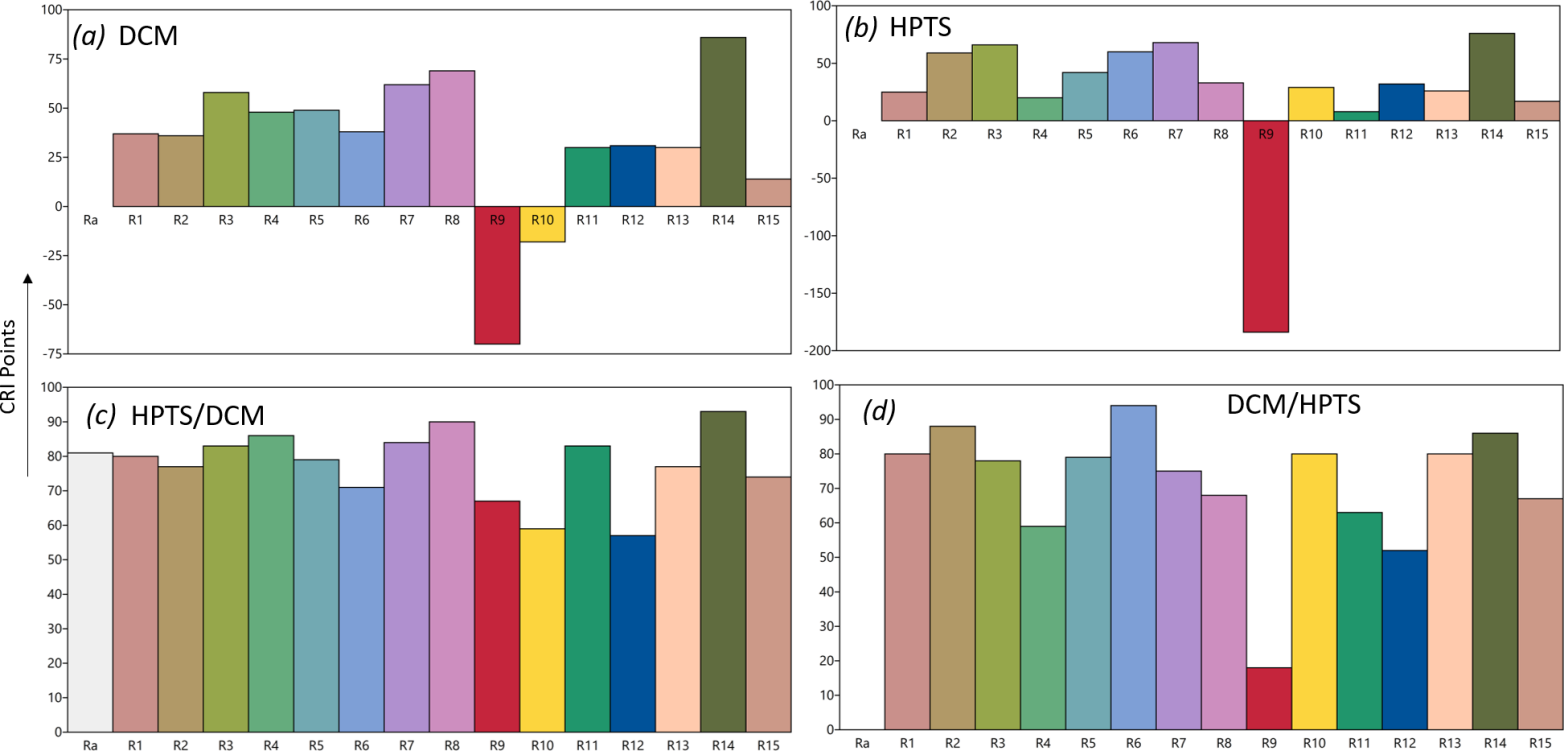
R-values charts for films containing: (a) DCM, (b) HPTS, (c) HPTS over DCM and (d) DCM over HPTS; measured under 450 nm LED illumination (440 mA, 3 V). All the films were placed in a remote-phosphor configuration at 0.3 cm from the LED chip.

The results showed that among all configurations, the HPTS/DCM/LED configuration is the one that yields a satisfactory emission in terms of CIE coordinates (0.33, 0.28). The corresponding colour rendering index (CRI or CIE *R*_a_) rating map, illustrated in Figure 8, showed that, with this configuration, the LED device can render colours with a precision comparable to that of a commercial YAG:Ce-based LED.

The CRI describes how truly the colour of an object is represented by a light source compared to a black-body source. Fifteen standard colour swatches (termed R1 through R15) plus the international standard colour rendering index *R*_a_ are represented in Figure 8. In the HPTS/DCM/LED configuration, all the colour indices values scored above 70 except R9, R10 and R12, which are assigned to strong saturated hues of red, yellow and blue. In the case of DCM/HPTS/LED configuration, R4 and R9 indices were comparatively low.

Red (R9) is a particularly difficult hue for YAG:Ce-based LEDs to render well. Red light is on the edge of the visible spectrum, where the human eye is less sensitive. A low R9 value leads to a low CRI. In the HPTS/DCM/LED configuration, we got a better CRI rating of 81. Note that a good daylight CRI rating is framed between 60 and 80 and an incandescent light bulb, considered a black-body radiator, exhibits a CRI of 100. Therefore, the HPTS/DCM/LED configuration exhibits a good CRI for lighting and display applications. The eyewitness account of a bright white light emitted by this system, suitable for indoor environments, is shown in the photograph in Figure 5c. In order to be considered as iso-energetic white, a LED device system needs to possess a correlated colour temperature (CCT) in the range of 2700–6000 K and the chromaticity coordinates falling on the black-body curve [20]. The apparent colour of the HPTS/DCM/LED configuration, determined by the CCT, was found to be 5409 K. This is assigned to a cool colour (bluish white) without infrared radiation, comparable to “vertical daylight” or an electronic flash. Table 2 reports all numeral values of photometric parameters for the HPTS/DCM/LED configuration in comparison to the DCM and HPTS films, as well as the DCM/HPTS/LED configuration.

**Table 2:** The luminous flux (Y2), colour rendering index (CRI), colour fidelity index *R*_f_, colour temperature (CCT), CIE colour coordinates (*x*,*y*) and optical efficacy of films based on DCM, HPTS; HPTS over DCM and DCM over HPTS; measured under 450 nm LED illumination (440 mA, 3 V). All the films were placed in a remote-phosphor configuration at 0.3 cm from the LED chip.

		DCM	HPTS	HPTS/DCM	DCM/HPTS

luminous flux Y2 (lm)		3.1	4.1	1.5	1.0
CRI (*R*_a_) [0–100]		33	7.60	81	44
*R*_f_		56	43	72	67
CCT (K)		—	—	5409	—
chromaticity	*x*	0.25	0.18	0.33	0.23
	*y*	0.14	0.28	0.28	0.26
optical efficacy (lm/W)		7.6	9.9	3.0	2.0

In the HPTS/DCM/LED configuration, the optical efficiency was measured at 3.0 lm/W, which is very low. It was found to be 34.9 lm/W for the phosphor-free LED and for a LED covered with a dye-free silicone film (silicone/LED). A value of 29.3 lm/W was measured for the LED covered with a silicone film without dye but containing only Zn-SLH (Zn-SLH-silicone/LED). Noted that, good phosphor-based converters have values above 150 lm/W. The reason for the lower values obtained was due to the configuration chosen for our measurements, i.e., the use of a high-output LED.

To sum, except their weak optical efficacy, the HPTS/DCM/LED configuration exhibited photometric parameters comparable to the ones of commercial YAG:Ce white LEDs [21].

## Conclusion

We have successfully developed luminescent composite films based on the mixture of a single-layered hydroxide and organic luminescent dyes. The organic dyes dicyanomethylene and pyranine were chosen according to their photoluminescent characteristics, which are excitation with blue light and an emission spectrum covering a wide range of the visible spectrum. The molecules were each dispersed in an inorganic matrix based on zinc hydroxyacetate single-layered hydroxide. In order to ensure good dispersion and avoid aggregation of the organic dyes, a composite preparation approach that kept the composite in its wet form prior to embedding in silicon second matrix was applied. The resulting films exhibit acceptable absolute quantum yields usable in LED devices. These films were placed on a 450 nm commercial LED in a remote-phosphor configuration to determine their photometric characteristics. The best results were obtained with the superposition of the pyranine film over that of dicyanomethylene. Both films were placed in a remote-phosphor configuration on top of a blue LED chip. The photometric parameters measured on this system [(CRI of 81, CCT of 5409 K, CIE coordinate of (0.33, 0.28)] were found to be very interesting for display applications. A bright white emission with cool colour temperature was obtained. Studies of the robustness of these luminescent films are underway with the aim to determine their mechanical and thermal stabilities. Furthermore, the stability of their optical properties will be investigated upon thermal and photonic stresses to demonstrate their ability for future applications.

## Experimental

### Materials

The hydrated zinc acetate Zn(CH_3_COO)_2_·2H_2_O (ZnAc_2_, 98% purity) was purchased from Alfa Aesar, absolute ethanol (EtOH, 100% purity), sodium hydroxide (NaOH), 4-(dicyanomethylene)-2-methyl-6-(4-dimethylaminostyryl)-4*H*-pyran (DCM, 98% purity) and trisodium 8-hydroxypyrene-1,3,6-trisulfonate (HPTS, 97% purity) were purchased from Sigma-Aldrich. The two-components Bluesil^TM^ RTV 141 A&B as silicon film precursors was purchased from Bluestar Silicones (Elkem France).

### Synthesis procedures

#### Preparation of the zinc single-layered hydroxide (Zn-SLH)

The general procedure involves the dissolution of 50 mmol ZnAc_2_ in 0.5 L absolute EtOH at 85 °C. After 1 h of agitation, the solution was cooled down to room temperature (RT) [14]. Then 12.22 mol of ultrapure milli-Q water was added to the solution under vigorous agitation. Hydrolysis occurs immediately and a white precipitate is formed progressively. After 1 h of ageing under magnetic stirring, the white product was centrifuged and washed several times with EtOH and stored after removing the supernatant solvent without drying.

A certain amount of the wet product was dried at 40 °C and weighed in order to determine the proportion of the dry extract (DE) to consider for the preparation of the luminescent hybrid material. The dry extract of the prepared Zn-SLH was evaluated at 16 wt %.

#### Preparation of wet luminescent hybrid materials (WLHM)

DCM and HPTS-containing Zn-SLH were prepared separately by impregnation before mixing the appropriate amount of each compound in the silicon film precursor in order to obtain the luminescent hybrid films.

In the impregnation procedure, each dye powder was previously dissolved in EtOH prior to mixing with the wet Zn-SLH. The composition of each mixture is given in Table 3. 1 wt % of dye in the DE-corresponding Zn-SLH was prepared in 15 mL of EtOH. After 24 h of impregnation under magnetic stirring at RT, the coloured wet mixture was recovered from the clear supernatant by centrifugation.

**Table 3:** Composition of each WLHM preparation.

	DCM	HPTS

concentration in EtOH (mg/mL)	1	1
wt % in WLHM	1	1
amount of wet HSL-Zn (*g*)	1	0.5
amount of DE HSL-Zn (16 wt % DE, g)	0.16	0.08
amount of dye (mg)	1.6	0.8
volume of dye (µL)	1600	800
total suspension volume adjustment (mL of EtOH)	15	15

#### Preparation of the white-emitting hybrid phosphor film (WEHP)

Each luminescent film was prepared by mixing the appropriate amount of each WLHM with the silicon polymer precursors, Bluesil^TM^ RTV 141 part A (90 wt %) and part B (10 wt %), with the consideration of their DE counterparts. Each mixture (WLHM and liquid silicon precursors) was prepared in a 3.5 cm diameter petri dish so as to obtain a film of 1.5 g weight and 3 mm thickness after drying in an oven overnight at 65 °C. The composition of WEHP preparation is given in Table 4.

**Table 4:** Composition of the white-emitting hybrid phosphor film (WEHP) preparation.

WLHM in the film		
	DCM/HSL-Zn	HPTS/HSL-Zn

wt % of DE for 1.5 g film	10%	2%
mass of DE for 1.5 g film (mg)	150	30
mass of WLHM to consider (g)	1	0.2

dye in the film		
	DCM	HPTS

wt % of Dye in the film	0.1	0.02
amount of dye in the film (mg)	0.15	0.03

### Characterization methods

The scanning electron microscopy (SEM) images of Zn-SLH powder were taken using a ZEISS Supra 55 FEG-VP instrument at 2MAtech (Clermont-Ferrand, France). The observations were carried out under high vacuum at 3 kV and using an Everhart–Thornley secondary-electron detector. Prior to observation, the sample was attached to adhesive carbon and then coated with Au.

The X-ray diffraction (XRD) pattern of Zn-SLH was recorded with a Philips Xpert Pro diffractometer operating with Cu Kα_1_ radiation (λ = 1.5406 Å) in the 2° < 2θ < 70° range with a scanning speed (2θ) of 0.03°/min.

Thermogravimetry and differential thermal analysis (TG-DTA) experiments were carried out on a SETARAME TG-DTA92 thermogravimetric analyser. The dried Zn-SLH sample was heated in air (25 mL/min) from 25 to 800 °C at a rate of 5 °C/min. The measurements were performed with ca. 15 mg of the sample in an alumina crucible.

Quantum yields (QY) were measured using a C9920-02G PL-QY measurement system from Hamamatsu. The setup consisted of a 150 W monochromatized Xe lamp, an integrating sphere (Spectralon coating, φ = 3.3 inch) and a high-sensitivity CCD camera. Photoluminescence excitation (PLE) spectra were obtained by exciting the composite films from 250 to 500 nm with 5 nm increment and measuring their absolute QY. The absolute photoluminescence (PL) QYs were calculated by combining the QY values with the absorption coefficient (also measured by the apparatus) to plot the excitation spectra.

The main photometric parameters of silicone/HSL-Dye films [photoluminescence (PL), luminous flux, correlated colour temperature (CCT), International Commission on Illumination (CIE) colour coordinates (*x,y*) and colour rendering Index (CRI)] were measured at room temperature in an integrating sphere with a diode array rapid analyser system (GL Optic integrating sphere GLS 500). In order to carry out the measurements, the film was placed on a 450 nm LED at a distance of 0.3 cm from the chip (OCC-X010S01A Optogan GmbH type, 1000 lm at 1 A, *T*: 3000–5300 K, Fwd current: 700–2100 mA) so that it completely covers the circular aperture (φ = 2.9 cm) of the cylindrical support of the LED. This set is then placed inside the integrating sphere at the opposite part of the spectrometer. Input current and voltage of 440 mA and 3 V respectively were applied before the measurement.
